# Supplementary material for: T cell exhaustion and a failure in antigen presentation drive resistance to the graft-versus-leukemia effect
Source: Nat Commun. 2020 Aug 24;11:4227. doi: 10.1038/s41467-020-17991-y (PMC7445289; doi:10.1038/s41467-020-17991-y)
Supplement: Supplementary file 1 — Supplemental Information [file 41467_2020_17991_MOESM1_ESM.pdf]

**Supplementary Figure 1. $T_{MH60}$ $T_{CM}$ progeny outcompete $T_{MH60}$ $T_{EM}$ progeny and the gating strategies for identifying mBC-CML and TetH60$^+$ cells.**

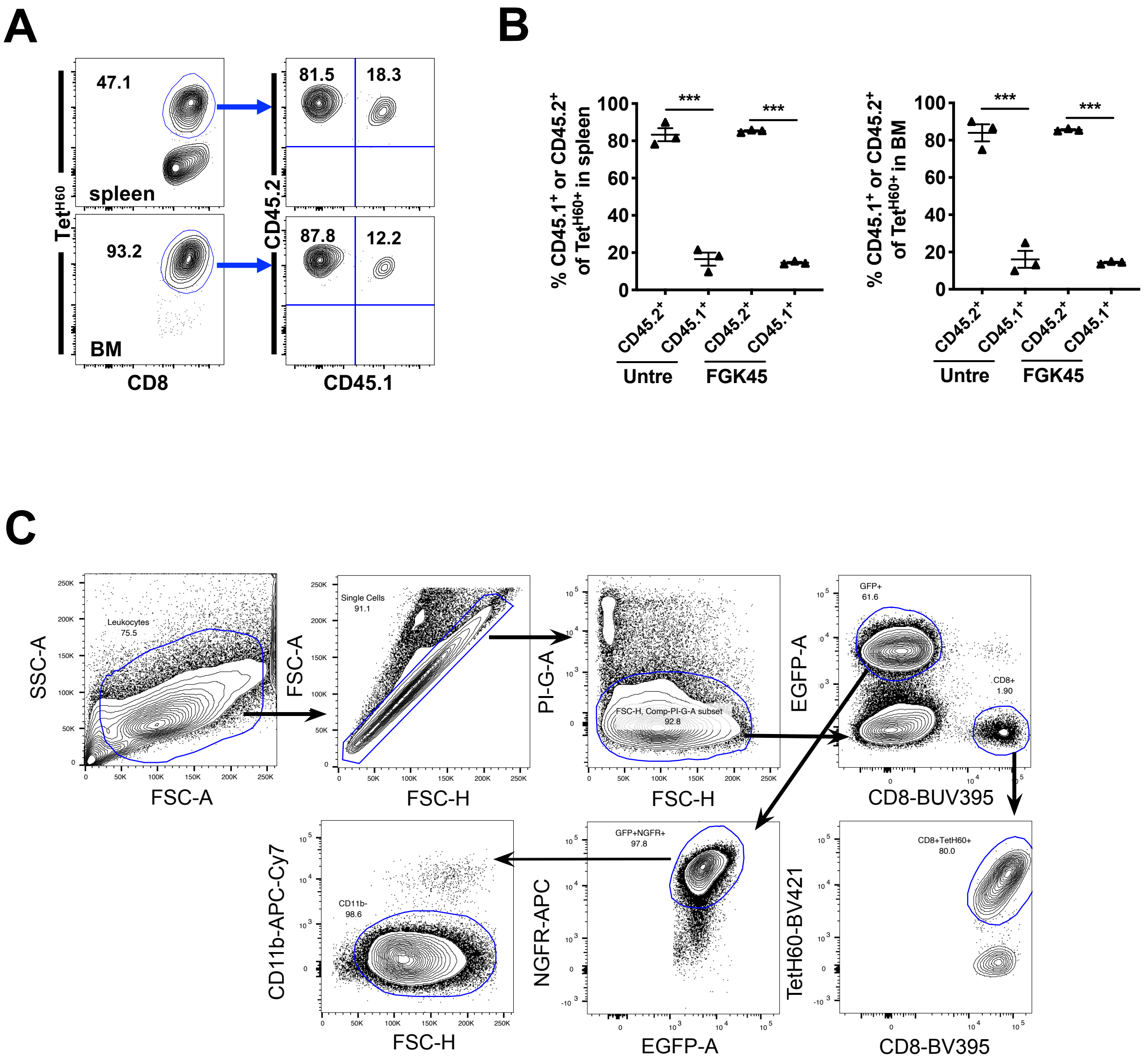

## Supplementary Figure Legends

**Supplementary Figure 1**. **Central memory $Tet^{H60+}$ cells dominate $Tet^{H60+}$ effector memory cells post-transplant and representative flow cytometry gating of BC-CML cells and $TetH60^{+}$ cells.** B6.H60 mice were irradiated and reconstituted with B6 BM, B6 $CD45.2^{+}CD44^{+}CD62L^{+}$ central memory ($T_{CM}$) $T_{MH60}$ (containing $5x10^{3}$ $Tet^{H60+}$ cells) and $CD45.1^{+}CD44^{+}CD62L^{-}$ effector memory ($T_{EM}$) $T_{MH60}$ (containing $5x10^{3}$ $Tet^{H60+}$ cells). One group of mice was also treated with FGK45 at the time of transplantation. Mice were sacrificed at day +7. Shown are representative flow cytometry (A) and the percentages of $T_{CM}$ and $T_{EM}$ derived progeny (B) in spleen and BM. Data are from one experiment (n=3). Unpaired Student two-sided t-test were used for statistical analysis. Bars are means +/- SEM. *$P \leq 0.0002$. (C). Represenative gating of $Tet^{H60+}$ and BC-CML cells among splenocytes post-transplant.

**Supplementary Figure 2 (in support of Figure 3). FGK45 and DEC-H60 independently contribute to Tet$^{H60+}$ T cell expansion without directly acting on BC-CML cells.**

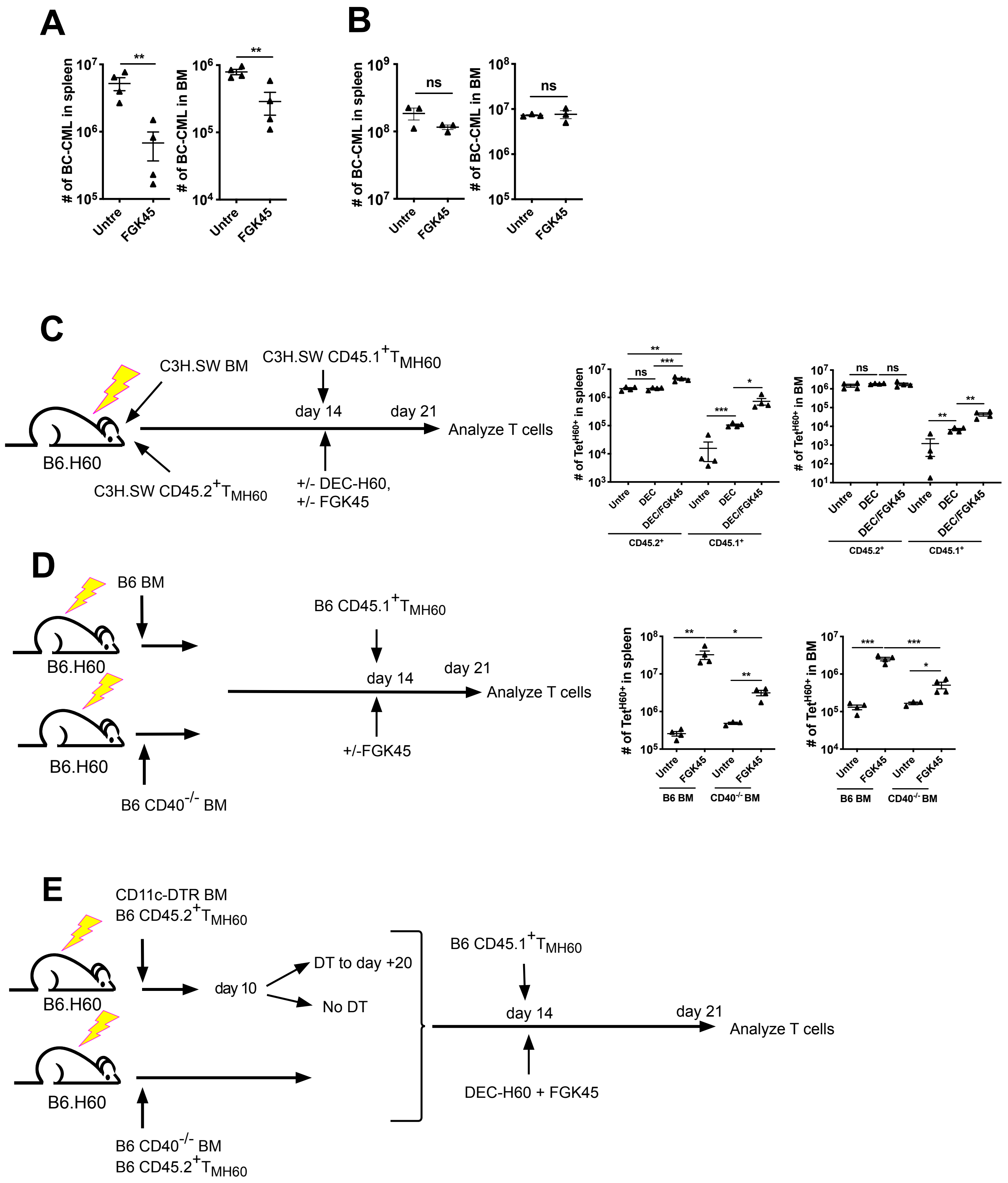

**Supplementary Figure 2 (in support of Figure 3). FGK45 and DEC-H60 independently contribute to $Tet^{H60+}$ cells expansion without directly acting on BC-CML cells.** (A) B6.H60 mice were irradiated and reconstituted with C3H.SW BM, C3H.SW $CD45.2^+$ $T_{MH60}$ with or without BC-CML. At day 14 post-transplant, fresh C3H.SW $CD45.1^+$ $T_{MH60}$ were infused. Mice did or did not receive FGK45 on day 14. Numbers of BC-CML cells in spleen and BM on day +21 are in (A). (B) B6 $RAG^{-/-}\gamma c^{-/-}$ mice were irradiated and transplanted with B6.H60 BC-CML with or without 100ug of FGK45 given on day 0. Mice were sacrificed at day +15 and BC-CML cells in spleen and BM were enumerated. (C) Irradiated B6.H60 mice were reconstituted with C3H.SW BM and C3H.SW $CD45.2^+$ $T_{MH60}$. On day +14 mice received DEC-H60 alone or with FGK45. Mice were sacrificed at day +21 and $Tet^{H60+}$ cells in spleen and BM were quantitated. (D) Irradiated B6.H60 mice were reconstituted with donor BM from B6 or B6 $CD40^{-/-}$ mice. At day +14 post-transplant, mice were injected with B6 $T_{MH60}$, with or without FGK45 treatment. Mice were sacrificed on day +21 and $T_{MH60}$ progeny in spleen and BM were quantitated. (E) Experimental design for donor DC depletion, in support of Fig. 3I. Panels in (A) are representative of 2 independent with 4 mice per group. Panels (B-D) are from one experiment with 3-4 mice per group. For all panels, an unpaired Student two-sided t-test was used for statistical analysis. Bars are means +/- SEM. *P< 0.05, **P< 0.01, ***P< 0.001.

**Supplementary Figure 3 in support of Figure 4. CID depletes iCasp9 K$^{b-/-}$ BC-CML cells**

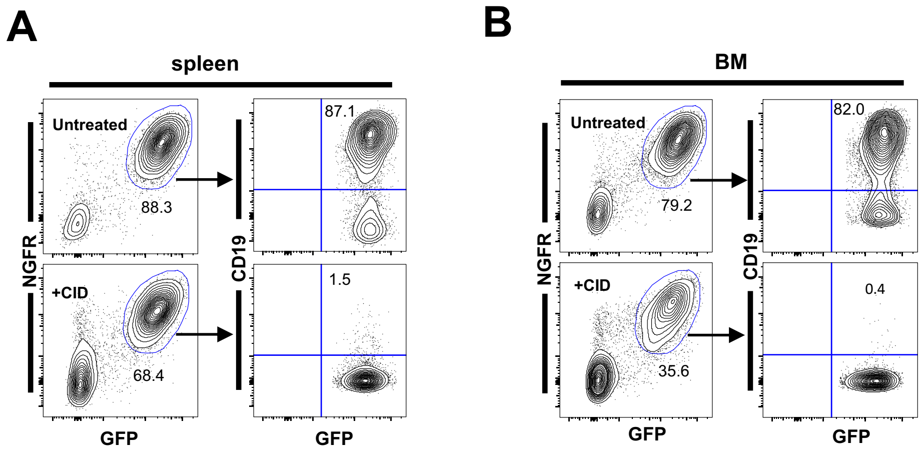

**Supplementary Figure 3 (in support of Figure 4). CID kills iCasp9 $K^{b-/-}$ BC-CML cells.** B6 mice were irradiated and reconstituted with C3H.SW BM and iCasp9 B6.H60$K^{b-/-}$ BC-CML which express CD19 linked to Casp9. One cohort was treated with CID (50μg /mice) every day from days +12 to days+14 post transplantation. At day +21 mice were sacrificed and spleen (A) and BM (B) were analyzed for the deletion of iCasp9$^{+}$ BC-CML based on expression of CD19.

**Supplementary Figure 4 (in support of Figure 5). Tet$^{H60+}$ cells post-transplant have features of T cell exhaustion.**

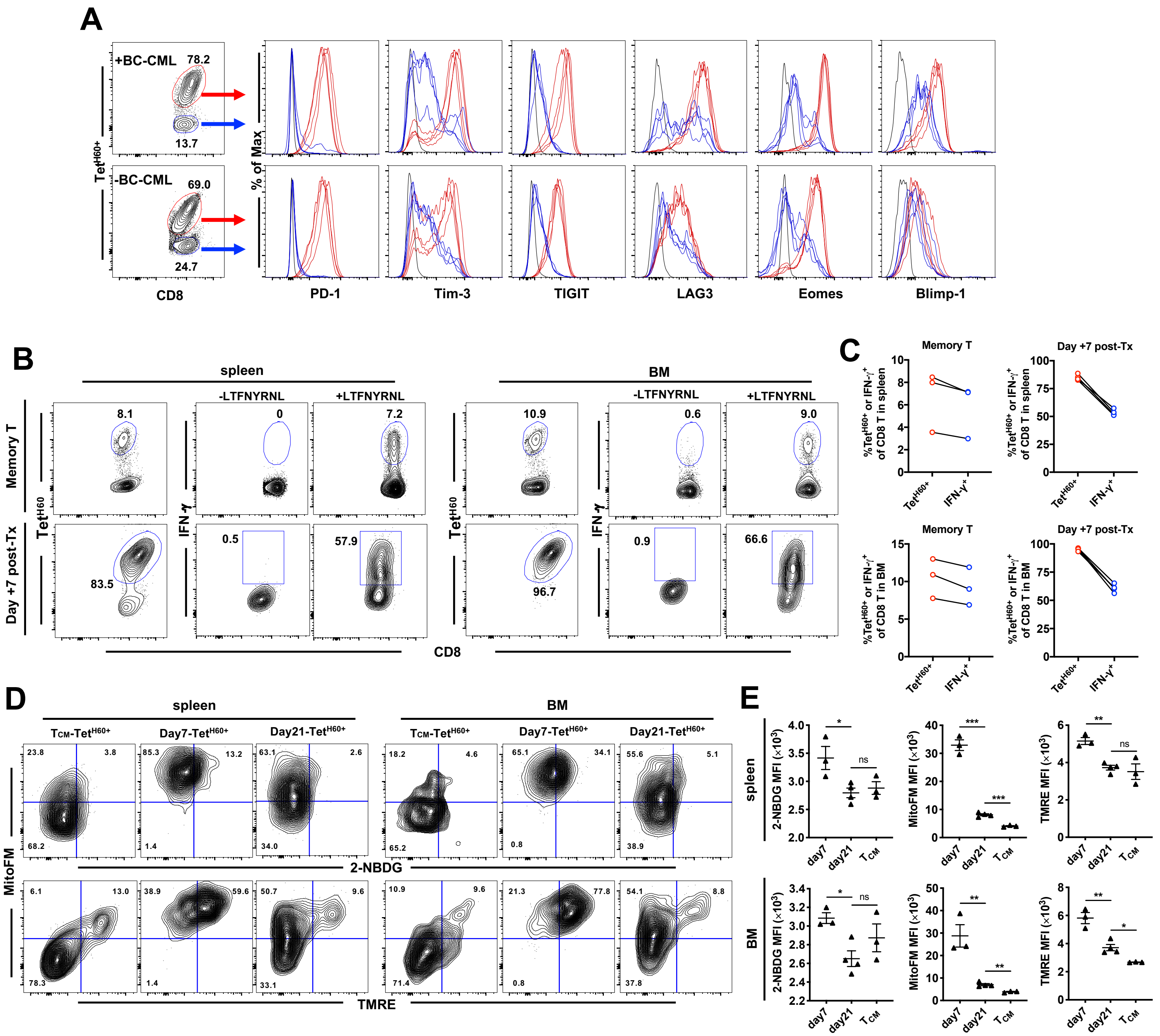

**Supplementary Figure 4 (in support of Figure 5). $Tet^{H60+}$ cells post-transplant have features of T cell exhaustion.** B6.H60 mice were irradiated and reconstituted with C3H.SW BM, C3H.SW $T_{MH60}$, with or without B6.H60 BC-CML. Mice were sacrificed 21-25 days post-transplantation for T cell analysis. (A) Expression of PD-1, Tim-3, TIGIT, LAG-3, Eomes and Blimp-1 on $Tet^{H60+}$ (red line) and $Tet^{H60-}$ (blue line) cells from spleen. (B and C) B6.H60 mice were irradiated and reconstituted with C3H.SW $T_{MH60}$. On day +7 splenocytes and BM cells were analyzed for $Tet^{H60}$ binding and IFN-$\gamma$ production in response to LTFNYRNL peptide stimulation by ICS. Fresh $T_{MH60}$ were also harvested from 3 memory mice, stained with $Tet^{H60}$ and stimulated to assess IFN-$\gamma$ production by ICS. Representative flow cytometry is in (B) and paired comparisons of the frequencies of $Tet^{H60+}$ and IFN-$\gamma^+$ cells are in (C). In a separate experiment, irradiated B6.H60 mice were reconstituted with C3H.SW BM and $T_{MH60}$. Fourteen days later a second cohort was transplanted. Seven days later (day +21 and +7 for the first and second cohorts, respectively), mice were sacrifice and cells were analyzed for 2-NBDG uptake and staining with MitoFM and TMRE. Fresh $T_{MH60}$ were also analyzed. Representative flow cytometry is in (D) and quantitation is in (E), gating on $CD8^+Tet^{H60+}$ cells. Note that day +7 $Tet^{H60+}$ cells had a high mitochondrial mass and stained brightly for TMRE whereas the majority of day +21 $Tet^{H60+}$ cells with a high mitochondrial mass had low TMRE staining. Panels (C) and (E) are from single experments with 3-4 mice per group. Unpaired Student two-sided t-tests were used for statistical analysis. Bars are mean values +/- SEM. *$P<0.05$, **$P<0.01$, ***$P<0.001$.

**Supplementary Figure 5 (in support of Figure 6). Effects of blocking PD-1, Tim-3 and TIGIT on GVL and the anti-H60 response.**

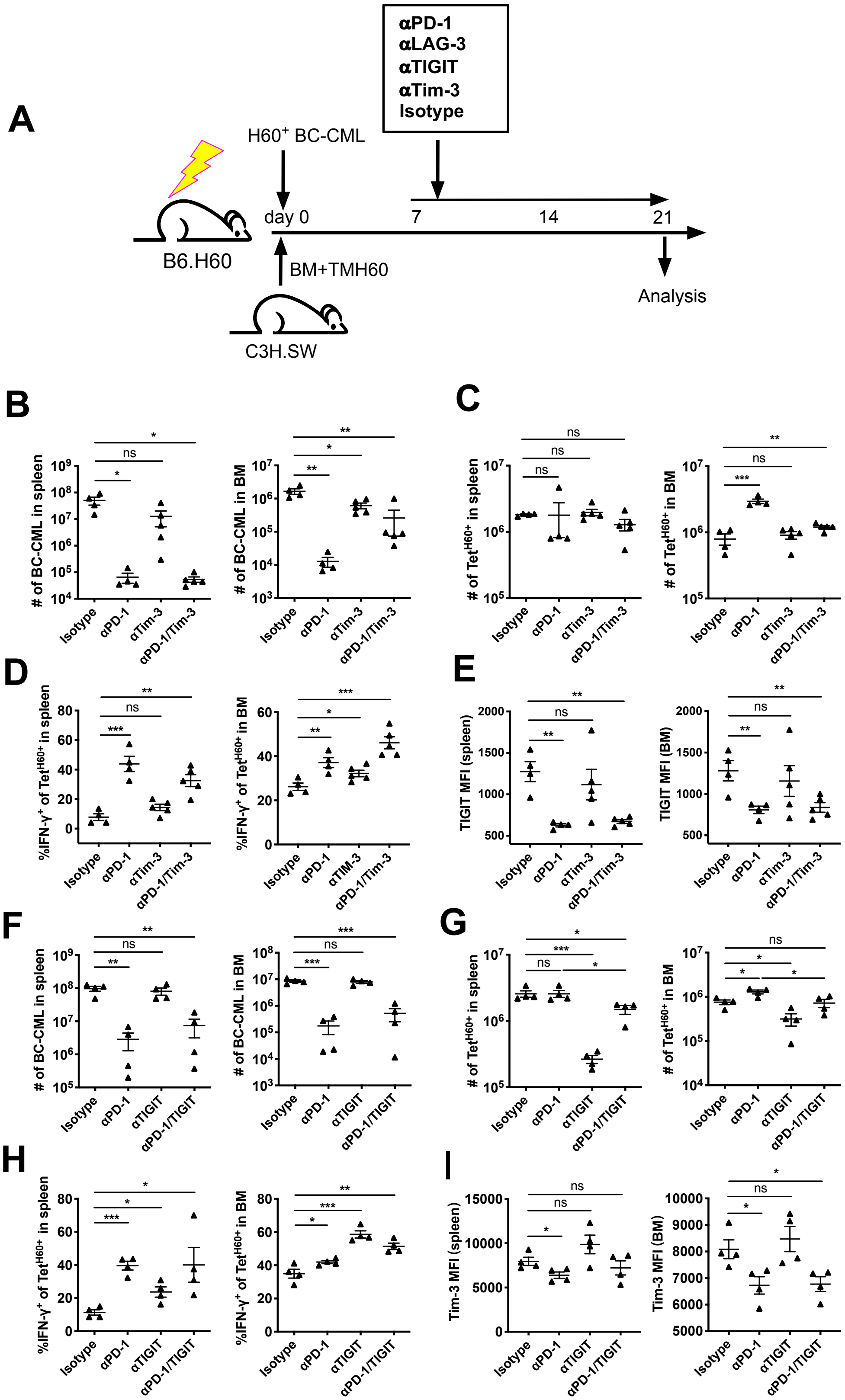

**Supplementary Figure 5 (in support of Figure 6). Effects of blocking PD-1, Tim-3 and TIGIT on GVL and the anti-H60 response.** (A), Experimental design. Irradiated B6.H60 mice were reconstituted with C3H.SW BM, B6.H60 BC-CML, and C3H.SW $T_{MH60}$. From days +7 to +19 post-transplantation, mice were treated with $\alpha$-PD1 (B-I), $\alpha$-Tim-3 (B-E) or $\alpha$-TIGIT (F-I) mAbs, alone or in combination with $\alpha$-PD1. Only $\alpha$-PD1, and not $\alpha$-Tim-3 or $\alpha$-TIGIT, reduced BC-CML numbers (B, F). Total number of $Tet^{H60+}$ in spleen and BM (C and G) and the frequencies of $Tet^{H60+}$ cells that produced IFN-$\gamma$ (D, H) are shown. TIGIT expression on $Tet^{H60+}$ cells with $\alpha$-PD1 (with or without $\alpha$-TIM-3), and Tim-3 expression (with and without $\alpha$-PD1 or $\alpha$-TIGIT) are shown in E and I, respectively. Experiments testing blockade of Tim-3 (4-5 mice per group) and TIGIT (4 mice per group) were from single experiments. For all panels, data were analyzed by an unpaired Student two-sided t-test. Bars are mean values +/- SEM. **P*<0.05; ***P*< 0.01 and ****P*<0.001.

**Supplementary Figure 6 (in support of Figure 7). Experimental design for mixed chimera experments in Figure 7 (panel A). Exclusive H60 cross-presentation diminishes T cell exhaustion (panels C and D).**

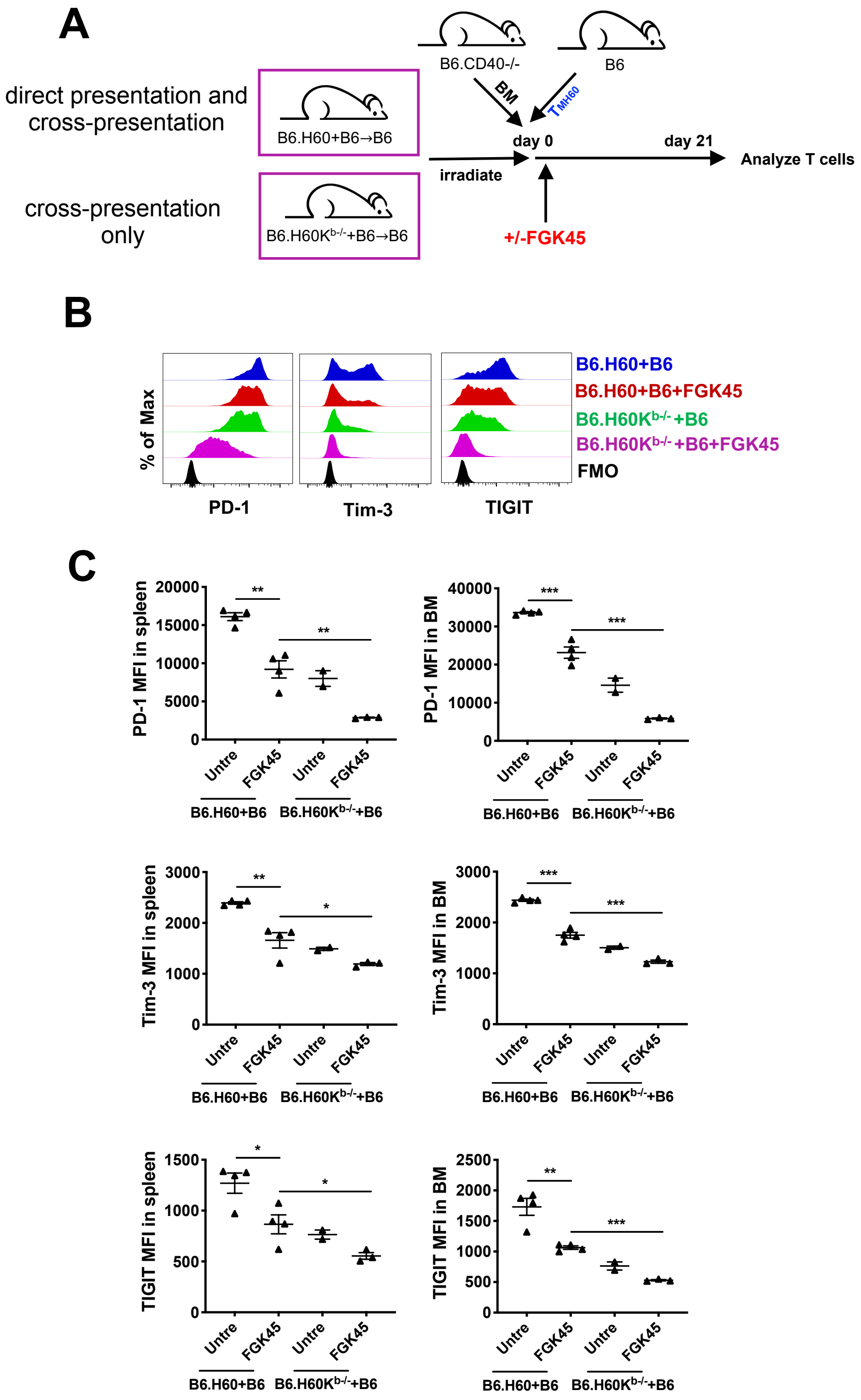

**Supplementary Figure 6 (in support of Figure 7). Exclusive H60 cross-presentation diminishes T cell exhaustion.** (A) Experimental design. B6.H60+B6→B6 and B6.H60$K^{b-/-}$ +B6→B6 BM chimeras were reirradiated and reconstituted with $CD40^{-/-}$ BM and B6 $T_{MH60}$ with or without FGK45. Spleen and BM cells were analyzed on day +21. Representative flow measurement of PD-1, Tim-3 and TIGIT expression on $Tet^{H60+}$ cells are in (B) and MFIs are quantitated in (C). Data are from one experiment (2-4 mice per group), unpaired Student two-sided t-test were used for statistical analysis. Bars are mean values +/- SEM. **P*<0.05; ***P*<0.01 and ****P*<0.001.

**Supplementary Figure 7 (in support of Figure 8). Effects of FGK45 given on day 0.**

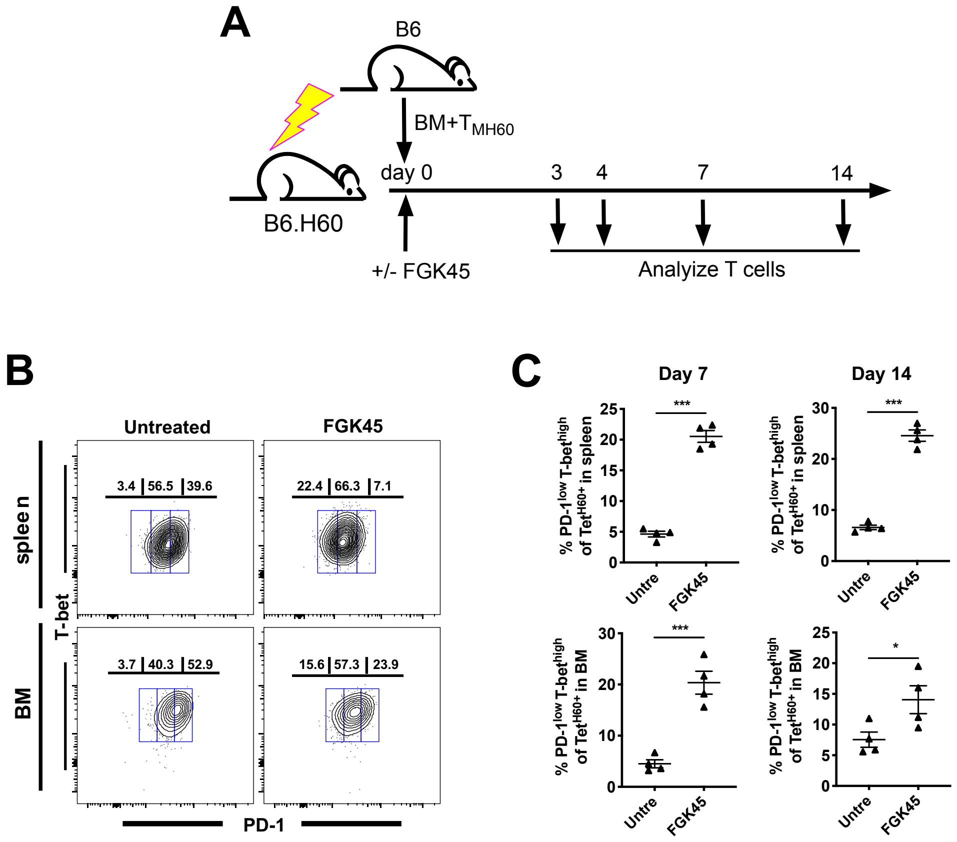

**Supplementary Figure 7 (in support of Figure 8). Impact of FGK45 given on day 0 on $T_{MH60}$ progeny infused on day 0.** (A) Experimental design. B6.H60 mice were irradiated and reconstituted with B6 BM and B6 $T_{MH60}$, with or without day 0 FGK45. Cohorts were sacrificed on days +3, +4, +7 and +14. Shown are representative flow cytometry of PD-1 versus T-bet expression on BM and spleen $Tet^{H60+}$ cells on day 7 (B) and quantitation in (C). Data are representative of two experiments (n=4 mice per group). Unpaired Student two-sided t-tests were used for statistical analysis. Bars are mean values +/- SEM. *P<0.05, and ***P<0.001.

**Supplementary Figure 8 (in support of Figure 9). Impact of FGK45 on Tet$^{H60-}$ CD8 cells.**

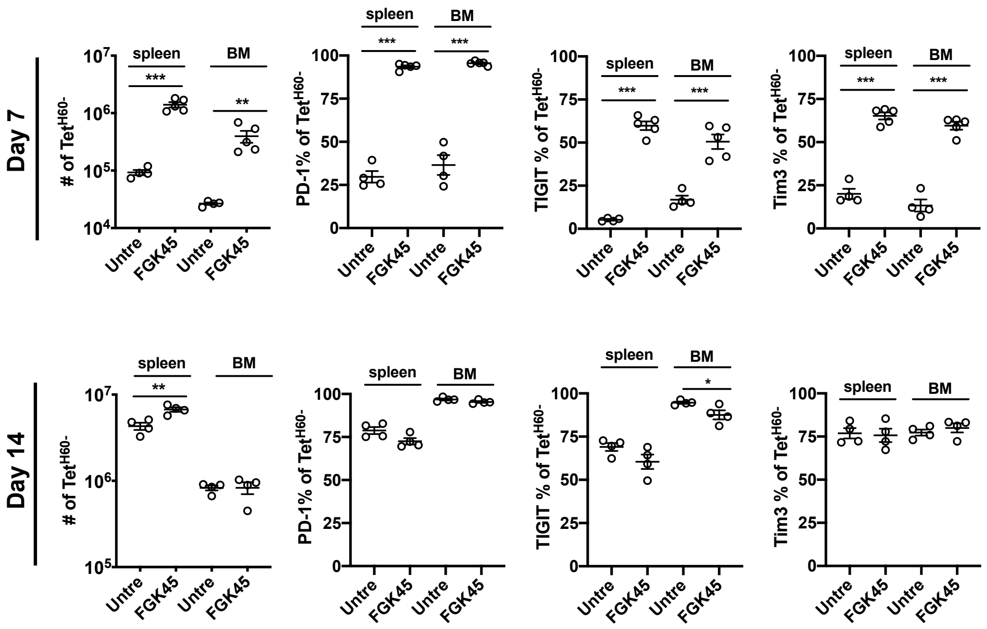

**Supplementary Figure 8 (in support of Figure 9). FGK45 increases the number of Tet$^{H60-}$ CD8 cells in C3H.SW→ B6.H60 recipients.** Irradiated B6.H60 mice were reconstituted with BM and CD8 cells from unmanipulated C3H.SW mice. Cohorts were sacrificed for analysis on days +7 and +14. Shown are numbers of Tet$^{H60-}$ CD8 cells and the percentages of those expressing PD-1, TIGIT and Tim-3. Data are representative of 2 experiments (n=4 or 5 mice per group). Unpaired Student two-sided t-tests were used for statistical analysis. Bars are mean values +/- SEM. *$P$<0.05; **$P$< 0.01 and ***$P$<0.001.

**Supplementary Figure 9 (in support of Figure 10). TOX and TCF-1 staining of control cells and $Tet^{H60+}$ and $Tet^{H60-}$ CD8 cells in bone marrow of transplanted mice.**

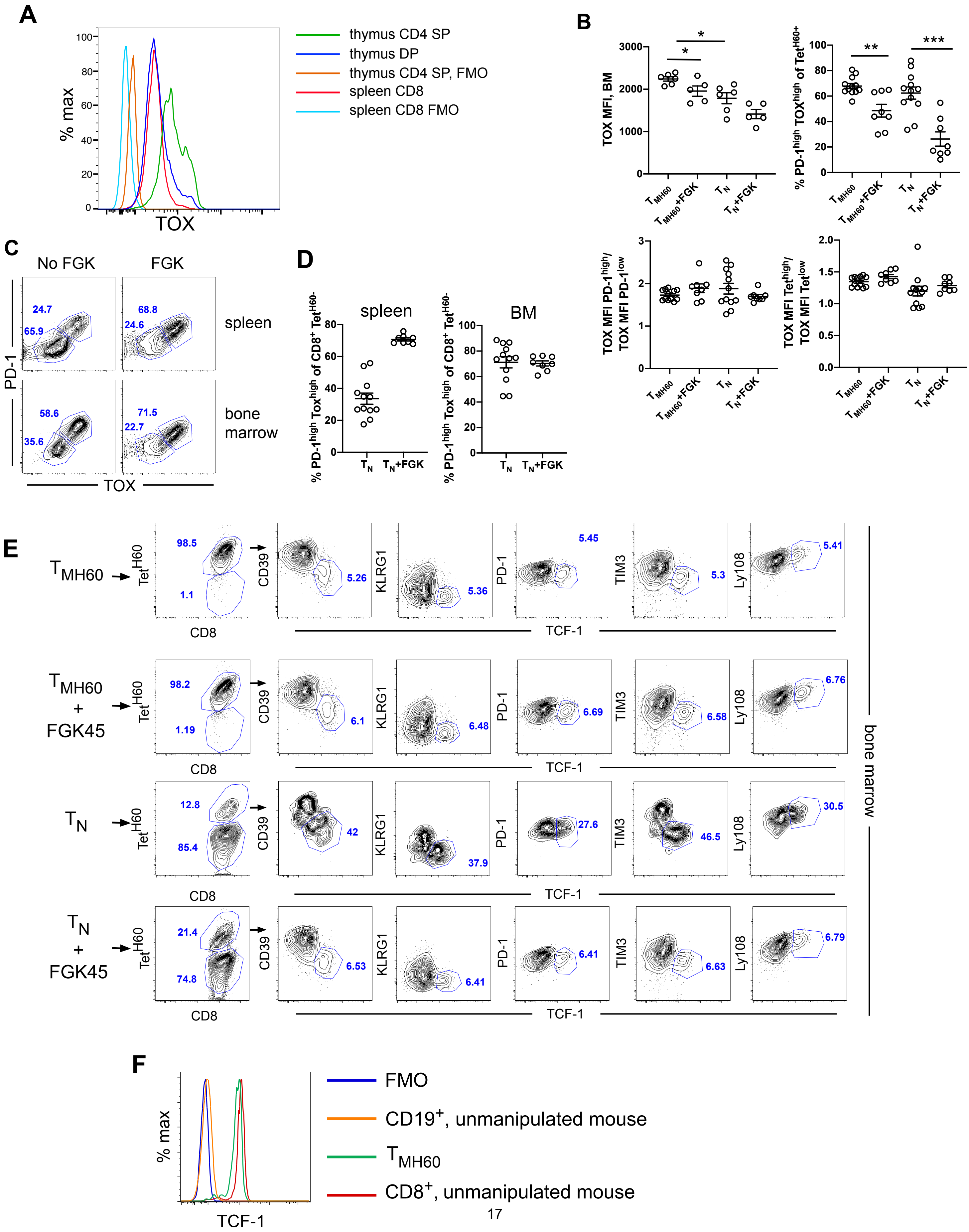

**Supplementary Figure 9 (in support of Figure 10). TOX and TCF-1 staining of control cells and $Tet^{H60+}$ and $Tet^{H60-}$ CD8 cells in bone marrow of transplanted mice.** (A) Staining of unmanipulated C3H.SW thymocytes and spleen cells for TOX. (B-E) Irradiated B6.H60 mice were reconstituted with C3H.SW BM and either C3H.SW $T_{MH60}$ or $10^6$ CD8 cells from unmanipulated C3H.SW mice ($T_N$). Recipients were sacrificed for analysis on day +8. Representative TOX staining of $Tet^{H60+}$ cells is in Figure 10. Data in (B) are gated on $Tet^{H60+}$ cells in BM. The TOX MFI was greater in $Tet^{H60+}$ PD-$1^{high}$ versus PD-$1^{low}$ and $Tet^{H60\ high}$ versus $Tet^{H60\ low}$ cells. FGK45 reduced the TOX MFI of $T_{MH60}$ progeny and the percentage of $T_N$ and $T_{MH60}$ $Tet^{H60+}$ progeny that were both PD-$1^{high}$ and $TOX^{high}$. (C) Representative TOX and PD-1 staining gating on $Tet^{H60-}$ cells in recipients of CD8 cells from unmanipulated donors; quantitation is in (D). (E) Expression of TCF-1 versus CD39, KLRG1, PD-1, Tim-3, and Ly-108 of $Tet^{H60+}$ cells from BM. (F) Blood $T_{MH60}$ and $CD8^+$ cells from unmanipulated mice were TCF-$1^+$ while $CD19^+$ B cells were TCF-$1^-$.TOX MFIs in (B) are representative of 2 independent experiments (5-6 mice per group). The remaining panels in (B) and (D) are pooled from 2 independent experiments (n=8 in the untreated group; n=12 in the FGK45 group). Unpaired Student two-sided t-tests were used for statistical analysis. Bars are mean values +/- SEM. *$P$<0.05; **$P$< 0.01 and ***$P$<0.001.
