# Supplementary material for: T cell exhaustion and a failure in antigen presentation drive resistance to the graft-versus-leukemia effect
Source: Nat Commun. 2020 Aug 24;11:4227. doi: 10.1038/s41467-020-17991-y (PMC7445289; doi:10.1038/s41467-020-17991-y)
Supplement: Supplementary file 3 — Reporting Summary [file 41467_2020_17991_MOESM3_ESM.pdf]

nature research

Corresponding author(s): Warren D Shlomchik

Last updated by author(s): 2020/06/10

Reporting Summary

Nature Research wishes to improve the reproducibility of the work that we publish. This form provides structure for consistency and transparency in reporting. For further information on Nature Research policies, see our Editorial Policies and the Editorial Policy Checklist.

Statistics

For all statistical analyses, confirm that the following items are present in the figure legend, table legend, main text, or Methods section.

n/a	Confirmed	
☐	☒	The exact sample size (n) for each experimental group/condition, given as a discrete number and unit of measurement
☐	☒	A statement on whether measurements were taken from distinct samples or whether the same sample was measured repeatedly
☐	☒	The statistical test(s) used AND whether they are one- or two-sided<br>*Only common tests should be described solely by name; describe more complex techniques in the Methods section.*
☒	☐	A description of all covariates tested
☒	☐	A description of any assumptions or corrections, such as tests of normality and adjustment for multiple comparisons
☐	☒	A full description of the statistical parameters including central tendency (e.g. means) or other basic estimates (e.g. regression coefficient) AND variation (e.g. standard deviation) or associated estimates of uncertainty (e.g. confidence intervals)
☐	☒	For null hypothesis testing, the test statistic (e.g. F, t, r) with confidence intervals, effect sizes, degrees of freedom and P value noted<br>*Give P values as exact values whenever suitable.*
☒	☐	For Bayesian analysis, information on the choice of priors and Markov chain Monte Carlo settings
☒	☐	For hierarchical and complex designs, identification of the appropriate level for tests and full reporting of outcomes
☒	☐	Estimates of effect sizes (e.g. Cohen's d, Pearson's r), indicating how they were calculated

Our web collection on statistics for biologists contains articles on many of the points above.

Software and code

Policy information about availability of computer code

Data collection	Flow cytometry data was collected with FACS Diva Software (version 9, BD Biosciences)
Data analysis	Flow cytometry data was analyzed with FlowJo version 10.5.3<br>All statistics were done using GraphPad prism 7.0 or 8.0

For manuscripts utilizing custom algorithms or software that are central to the research but not yet described in published literature, software must be made available to editors and reviewers. We strongly encourage code deposition in a community repository (e.g. GitHub). See the Nature Research guidelines for submitting code & software for further information.

Data

Policy information about availability of data

All manuscripts must include a data availability statement. This statement should provide the following information, where applicable:

- Accession codes, unique identifiers, or web links for publicly available datasets
- A list of figures that have associated raw data
- A description of any restrictions on data availability

The data that support the findings of this study are available from the corresponding author upon reasonable request.

Field-specific reporting

Please select the one below that is the best fit for your research. If you are not sure, read the appropriate sections before making your selection.

- [x] Life sciences
- [ ] Behavioural & social sciences
- [ ] Ecological, evolutionary & environmental sciences

For a reference copy of the document with all sections, see nature.com/documents/nr-reporting-summary-flat.pdf

Life sciences study design

All studies must disclose on these points even when the disclosure is negative.

Sample size	Sample sizes were chosen based on prior experience in this model and similar systems. These sample sizes are sufficient in that we were interested in the most biologically significant changes. We report a P value for every data comparison and make no claims where there were not significant P values.
Data exclusions	No data were excluded from the analyses.
Replication	Independent repeats were performed as stated in each figure legend. All attempts at replication were successful.
Randomization	All samples were assigned to groups randomly.
Blinding	No blinding, as the same investigator performed most experiments and analyzed the data

Reporting for specific materials, systems and methods

We require information from authors about some types of materials, experimental systems and methods used in many studies. Here, indicate whether each material, system or method listed is relevant to your study. If you are not sure if a list item applies to your research, read the appropriate section before selecting a response.

Materials & experimental systems

n/a	Involved in the study	
☐	☒	Antibodies
☐	☒	Eukaryotic cell lines
☒	☐	Palaeontology and archaeology
☐	☒	Animals and other organisms
☒	☐	Human research participants
☒	☐	Clinical data
☒	☐	Dual use research of concern

Methods

n/a	Involved in the study	
☒	☐	ChIP-seq
☐	☒	Flow cytometry
☒	☐	MRI-based neuroimaging

Antibodies

Antibodies used

Antibody/Catlog number/reagnet Source/Clone/dilution
mCD44 #103005 Biolegend IM7 1/400 dilution
mCD62L #104438 Biolegend MEL-14 1/800 dilution
mCD45.1 #110714 Biolegend A20 1/200 dilution
mCD45.2 #109806 Biolegend 104 1/200 dilution
mCD45.2 #109830 Biolegend 104 1/200 dilution
mCD11b #101224 Biolegend M1/70 1/400 dilution
mH2-Kb #116514 Biolegend AF6-88.5 1/400 dilution
mI-A/I-E #107639 Biolegend M5/114.15.2 1/400 dilution
mKLRG1 #138427 Biolegend 2F1 1/200 dilution
mPD-1 #135216 Biolegend 29F.1A12 1/400 dilution
mPD-1 #135214 Biolegend 29F.1A12 1/200 dilution
mTim-3 #119721 Biolegend RMT3-23 1/400 dilution
mTim-3 #119704 Biolegend RMT3-23 1/400 dilution
mLAG3 Lab of Dario A.A. Vignali 4-10-C9 1/200 dilution
mTIGIT #142106 Biolegend 1G9 1/200 dilution
mTIGIT #142104 Biolegend 1G9 1/200 dilution
mCD8 #100714 Biolegend 53-6.7 1/400 dilution
mCD8 #563786 BD bioscience 53-6.7 1/200 dilution
mCD137(4-1BB) #106110 Biolegend 17B5 1/200 dilution
mOX-40 #119419 Biolegend OX-86 1/200 dilution

mIL-2Ra #102042 Biolegend PC61 1/200 dilution
mGITR #558140 BD Biosciences DTA-1 1/200 dilution
mIFN-g #554412 BD Biosciences XMG1.2 1/100 dilution
mTNF-a #506324 Biolegend MP6-XT22 1/100 dilution

mEomes #12-4875-82 Thermo Fisher Scientific Dan11mag 1/100 dilution
mT-bet #12-5825-82 Thermo Fisher Scientific 4B10 1/100 dilution
mTOX #12-6502-82 Thermo Fisher Scientific TXRX10 1/100 dilution
mCD39 #46-0391-80 Thermo Fisher Scientific 24DMS1 1/200 dilution
mBlimp-1 #150004 Biolegend 5E7 1/100 dilution
mICAM-1 #116120 Biolegend YN1/1.7.4 1/400 dilution
mLy108 #134609 Biolegend 330-AJ 1/400 dilution
mTCF-1 #564217 BD Biosciences S33-966 1/100 dilution
PD-1 blocking antibody #329945 Biolegend RMP1-14
LAG-3 blocking antibody Lab of Dario A.A. Vignali C9B7W
Tim3 blocking antibody #119711 Biolegend RMT3-23
TIGIT blocking antibody Genentech 10A7
mCD8a laboratory prepared TIB105 1/400 dilution
Fixable Viability Dye eFluor780 Thermo Fisher Scientific 1/1000 dilution

Validation

mCD44 validated by Flow Cytometry (FC)using C57BL/6 mouse splenocytes (Biolegend website)
mCD62L validated by FC using C57BL/6 mouse splenocytes (Biolegend website)
mCD45.1 validated by FC using SJL mouse splenocytes (Biolegend website)
mCD45.2 validated by FC using C57BL/6 mouse splenocytes (Biolegend website)
mCD11b validated by FC using C57BL/6 mouse bone marrow cells (Biolegend website)
mH2-Kb validated by FC using C57BL/6 mouse splenocytes (Biolegend website)
mI-A/I-E validated by FC using C57BL/6 mouse splenocytes (Biolegend website)
mKLRG1 validated by FC using C57BL/6 mouse splenocytes (Biolegend website)
mPD-1 validated by FC using Con-A stimulated C57BL/6 mouse splenocytes (Biolegend website)
mTim-3 validated by FC using Mouse Tim-3 transfected cells (Biolegend website)
mLAG3 (clone:4-10-C9) validated by Dario A.A. Vignali' lab and utilized in recent publications (Sci Immunol 2017 Mar 31;2 (9):eaah4569. doi: 10.1126/sciimmunol.aah4569)
mTIGIT validated by FC using Con-A stimulated C57BL/6 mouse splenocytes (Biolegend website)
mCD137(4-1BB) validated by FC using ConA+IL-2 (3 days) stimulated C57BL/6 splenocyte (Biolegend website)
mOX-40 validated by FC using ConA+IL-2 (3 days) stimulated C57BL/6 splenocyte (Biolegend website)
mIL-2Ra validated by FC using Con A-stimulated (3 days) Balb/c splenocytes (Biolegend website)
mGITR validated by FC using BALB/c splenocytes (BD Biosciences website)
mIFN-g validated by FC using aCD3/CD28-stimulated C57BL/6 mouse splenocytes (BD Biosciences website)
mTNF-a validated by FC using PMA + Ionomycin-stimulated C57BL/6 mouse splenocytes (Biolegend website)
mEomes validated by FC using C57BL/6 mouse splenocytes (Thermo Fisher Scientific website)
mT-bet validated by FC using normal human peripheral blood cell (Thermo Fisher Scientific website)
mTOX validated by FC using C57BL/6 mouse thymocytes (Thermo Fisher Scientific website) mCD39 validated by FC using C57BL/6 mouse splenocytes (Thermo Fisher Scientific website)
mBlimp-1 Biolegend 5E7 using LPS stimulated C57BL/6 mouse splenocytes (Biolegend website)
mICAM-1 validated by FC using C57BL/6 mouse splenocytes (Biolegend website)
mLy108 validated by FC using C57BL/6 mouse splenocytes (Biolegend website)
mTCF-1 validated by FC using Mouse splenic leucocytes (BD Biosciences website)
PD-1 blocking antibody Blocking application is quality tested via Biolegend, and has cited in the literature (Takamura S, et al. 2010. J. Immunol. 184:4696) for this application
LAG-3 blocking antibody (clone:C9B7W) Blocking application validated previous by Lab of Dario A.A. Vignali (Cancer Res. 2012 Feb 15; 72(4): 917–927)
Tim3 blocking antibody Blocking application is quality tested via Biolegend, and has cited in the literature (Blood. 2007 Oct 1; 110(7): 2565–2568) for this application
TIGIT blocking antibody validated previously by Genentech (Cancer Cell. 2014 Dec 8;26(6):923-937. doi: 10.1016/j.ccell.2014.10.018. Epub 2014 Nov 26).
mCD8a this laboratory prepared validated previously (J Immunol. 2011 Aug 15;187(4):1653-63. doi: 10.4049/jimmunol.1100311. Epub 2011 Jul 18).
mCD8 from Biolegend or BD bioscience validated by FC using mouse splenocytes (Biolegend website or BD Biosciences website)

Eukaryotic cell lines

Policy information about cell lines

Cell line source(s)

Our original descriptions of the blast crisis chronic phase chronic myelogenous leukemia (BC-CML) induction were described in Journal of immunology 187, 1653-1663, doi:10.4049/jimmunol.1100311 (2011). These are not cell lines propagated in

	culture but can only be passaged in mice as described in the paper. We created additional new BC-CML lines in the course of our work.
Authentication	BC-CML were not authenticated but utilized after 2 round passage from mice to mice. Premorbid mice were sacrificed, and splenocytes were frozen. These cells were passaged in sublethally irradiated mice, from which splenocytes were harvested and frozen (secondary mice). After that, frozen splenocytes from secondary mice were used as the BC-CML cells.
Mycoplasma contamination	This is not applicable as these cell lines are not kept in culture.When our original BC-CML lines were moved from Yale to Pitt, they were tested for mycoplasma and those tests were negative.
Commonly misidentified lines (See ICLAC register)	No commonly misidentified cell lines were used.

Animals and other organisms

Policy information about studies involving animals; ARRIVE guidelines recommended for reporting animal research

Laboratory animals	6-8 week old B6.SJL-Ptprca Pepcb/BoyJ (CD45.1, H-2b), B6 CD11c-DTR, B6 CD40-/-, B6 beta-2-microglobulin-deficient (b2M-/-), B6 ubiquitin-GFP transgenic and B6 RAG-/- and C3H.SW (H-2b; H60-) mice were purchased from Jackson Labs (JAX) and were bred at the University of Pittsburgh (Pitt). 6-8 week old B6 Kb-/- mice were purchased from Taconic. 6-8 week old OT-1 RAG1-/- and B6-Rag2tm1Fwa Il2rgtm1Wjl (RAG-/-gc-/-) mice were provided by Fadi Lakkis (Pitt). B6.H60 mice were originally obtained from Derry Roopenian (JAX) and were bred at Pitt. B6.H60Kb-/- and B6.H60-CD11c.DTR mice were generated at Pitt. C3H.SW CD45.1+ mice were generated by crossing C3H.SW mice to B6.CD45.1+ mice (>10 generations). Mice either male or female were used in the study.
Wild animals	This study did not involve wild animals
Field-collected samples	This study did not involve samples collected from field
Ethics oversight	All animals were used according to protocols approved by Institutional Animal Care and Use Committee of the University of Pittsburgh.

Note that full information on the approval of the study protocol must also be provided in the manuscript.

Flow Cytometry

Plots

Confirm that:

- [x] The axis labels state the marker and fluorochrome used (e.g. CD4-FITC).
- [x] The axis scales are clearly visible. Include numbers along axes only for bottom left plot of group (a 'group' is an analysis of identical markers).
- [x] All plots are contour plots with outliers or pseudocolor plots.
- [x] A numerical value for number of cells or percentage (with statistics) is provided.

Methodology

Sample preparation	Spleen and Bone marrow samples from leukemia bearing mice were processed into single cells through 70 micron filter, and additionally lysed with ACK lysis buffer to remove red blood cells.
Instrument	BD Biosciences FACSAria sorting, FACSAria for analyzing
Software	FlowJo 9 and 10 for data analysis.
Cell population abundance	The purity of the sorted cells was determined after FACS acquisition or magnetic beads isolation.
Gating strategy	To analyze cells a preliminary FSC/SSC gate were utilized to gate on the morphology of leukocytes. Subsequently, a singles gate (FSC-A vs FSC-H) was used to exude the doublets followed by a viability gate (PI, Fixable dye) to exclude dead cells. From this population, relevant gating strategies for each cell type are described.

- [x] Tick this box to confirm that a figure exemplifying the gating strategy is provided in the Supplementary Information.
